# The Main Progress of Perovskite Solar Cells in 2020–2021

**DOI:** 10.1007/s40820-021-00672-w

**Published:** 2021-07-07

**Authors:** Tianhao Wu, Zhenzhen Qin, Yanbo Wang, Yongzhen Wu, Wei Chen, Shufang Zhang, Molang Cai, Songyuan Dai, Jing Zhang, Jian Liu, Zhongmin Zhou, Xiao Liu, Hiroshi Segawa, Hairen Tan, Qunwei Tang, Junfeng Fang, Yaowen Li, Liming Ding, Zhijun Ning, Yabing Qi, Yiqiang Zhang, Liyuan Han

**Affiliations:** 1 State Key Laboratory of Metal Matrix Composites, School of Material Science and Engineering, Shanghai Jiao Tong University, Shanghai, 200240 People’s Republic of China; 2 Key Laboratory for Advanced Materials and Feringa Nobel Prize Scientist Joint Research Centre, Shanghai Key Laboratory of Functional Materials Chemistry, Joint International Research Laboratory of Precision Chemistry and Molecular Engineering, School of Chemistry and Molecular Engineering, East China University of Science and Technology, Meilong Road 130, Shanghai, 200237 People’s Republic of China; 3 Wuhan National Laboratory for Optoelectronics, Huazhong University of Science and Technology, Luoyu Road 1037, Wuhan, 430074 People’s Republic of China; 4 College of Materials Science and Engineering, Nanjing University of Science and Technology, Nanjing, 210094 People’s Republic of China; 5 Beijing Key Laboratory of Novel Thin-Film Solar Cells and State Key Laboratory of Alternate Electrical Power System With Renewable Energy Sources, North China Electric Power University, Beijing, 102206 People’s Republic of China; 6 Department of Microelectronic Science and Engineering, Ningbo University, Zhejiang, 315211 People’s Republic of China; 7 College of Chemical Engineering, Jiangsu Key Lab of Biomass-Based Green Fuels and Chemicals, Nanjing Forestry University, Nanjing, 210037 People’s Republic of China; 8 College of Chemistry and Molecular Engineering, Qingdao University of Science and Technology, Qingdao, 266042 People’s Republic of China; 9 Special Division of Environmental and Energy Science, Komaba Organization for Educational Excellence (KOMEX), College of Arts and Sciences, University of Tokyo, Tokyo, 153-8902 Japan; 10 National Laboratory of Solid State Microstructures, Jiangsu Key Laboratory of Artificial Functional Materials, College of Engineering and Applied Sciences, Nanjing University, Nanjing, 210093 People’s Republic of China; 11 College of Information Science and Technology, Jinan University, Guangzhou, 510632 People’s Republic of China; 12 School of Physics and Electronic Science, Engineering Research Center of Nanophotonics and Advanced Instrument, Ministry of Education, East China Normal University, Shanghai, 200062 People’s Republic of China; 13 Laboratory of Advanced Optoelectronic Materials, College of Chemistry, Chemical Engineering and Materials Science, Soochow University, Suzhou, 215123 People’s Republic of China; 14 Center for Excellence in Nanoscience, Key Laboratory of Nanosystem and Hierarchical Fabrication, National Center for Nanoscience and Technology, Beijing, 100190 People’s Republic of China; 15 School of Physical Science and Technology, ShanghaiTech University, 100 Haike Road, Shanghai, 201210 People’s Republic of China; 16 Energy Materials and Surface Sciences Unit (EMSSU), Okinawa Institute of Science and Technology Graduate University (OIST), Okinawa, 904-0495 Japan; 17 School of Materials Science and Engineering, Henan Institute of Advanced Technology, Zhengzhou University, Zhengzhou, 450001 People’s Republic of China

**Keywords:** Perovskite solar cells, Stability, Solar module, Perovskite-based tandem devices, Lead-free perovskite

## Abstract

Perovskite solar cells (PSCs) emerging as a promising photovoltaic technology with high efficiency and low manufacturing cost have attracted the attention from all over the world. Both the efficiency and stability of PSCs have increased steadily in recent years, and the research on reducing lead leakage and developing eco-friendly lead-free perovskites pushes forward the commercialization of PSCs step by step. This review summarizes the main progress of PSCs in 2020 and 2021 from the aspects of efficiency, stability, perovskite-based tandem devices, and lead-free PSCs. Moreover, a brief discussion on the development of PSC modules and its challenges toward practical application is provided.

## Highlights

- Recent progress of efficiency and long-term stability for perovskite solar cells, and the development of perovskite-based tandem solar cells are described.
- The progress of lead-free perovskite solar cells and their potential for industrial production are discussed in detail.
- The current status, ongoing challenges, and the future outlooks of perovskite solar cells are highlighted.

## Graphical abstract

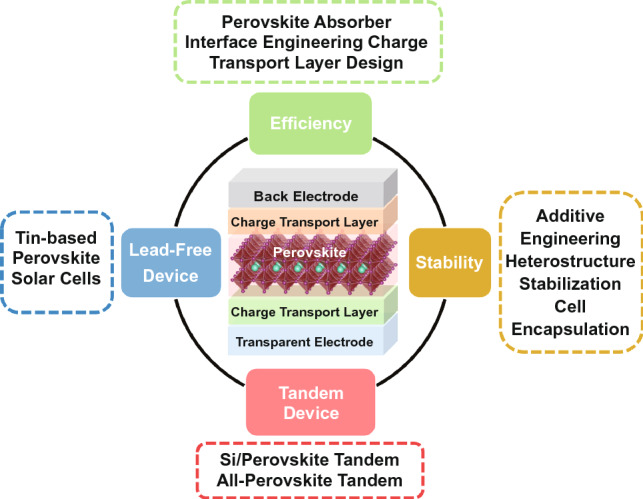

## Introduction

Perovskite solar cells (PSCs) have become a promising thin-film photovoltaic (PV) technology due to the high light-absorption coefficient, long carrier diffusion length, and solution processibility of metal halide perovskite materials [1–5]. Currently, the highest power conversion efficiency (PCE) of PSCs has reached 25.5% [6], exceeding the record efficiency of copper indium gallium selenium (CIGS) solar cells and approaching that of crystalline-Si solar cells. Moreover, the scale-up deposition techniques for perovskites and charge transport layers promote the development of large-area perovskite solar modules, including doctor-blade coating, slot-die coating, screen printing, and spray deposition strategies. Recently, a certified efficiency of about 18% has been reported for an over 800 cm^2^ PSC sub-module [6], indicating large potential for practical use. On the other hand, perovskite-based tandem solar cells with a theoretical PCE beyond the limit of single-junction PSCs also make a huge progress owing to the reduction of defect density and increase of carrier diffusion length in both wide-bandgap and narrow-bandgap perovskite absorbers [7–9]. By now, the highest certified efficiency of the perovskite-based tandems has increased to over 29% [10].

Besides the efficiency progress, the long-term stability of PSCs against damp, light, and heat also improved significantly in recent years, which could be attributed to the construction of diffusion barrier against ion migration, additive engineering, design of chemically inert carbon-based electrodes, and development of cell encapsulation technique to reduce the lead leakage from a broken PSC module [11–18]. It was demonstrated that the printable PSCs had passed the most popular international standards of IEC61215:2016 for mature PV technology [19].

With the continuous progress of PSCs toward commercialization, exploiting eco-friendly lead-free perovskite materials has also become a hot research topic in this field in view of the toxicity of Pb element in Pb-containing PSCs giving rise to the concern of environmental pollution [20–23]. So far, the highest certified efficiency of lead-free PSCs has reached 11.22%, enabled by minimizing the defect density in tin halide perovskite films via a template-growth deposition method [24].

In this review, we summarize the representative works on PSCs published by worldwide research groups in 2020**–**2021 from the aspects of efficiency, stability, perovskite-based tandem solar cells, and the development of lead-free PSCs. In addition, we point out the future challenges on realizing the commercialization of PSCs, and then give a brief outlook on the possible research topics at the next stage.

## Efficiency

PSCs are usually composed of perovskite absorbers, charge transport layers and counter electrodes. The energy loss in the bulk and interface of perovskites layers, and the charge extraction and transportation process in device play a critical role in determining the efficiency of PSCs. Therefore, improving the crystal quality of perovskite films, suppressing the non-radiative recombination at interface, and rational design of the charge transport layers are expected to further improve the device efficiency.

### Perovskite Absorbers

In 2020–2021, the research activities on perovskite layer mainly focus on stabilizing the formamidinium lead iodide (FAPbI_3_) perovskite phase with wide absorption range and long carrier lifetime to boost the short-circuit current density (*J*_SC_) and open-circuit voltage (*V*_OC_) of PSCs [25, 26]. Besides, exploiting the single-crystal device with very low defect density has also been reported for realizing high-performance PSCs.

**Fig. 1:**
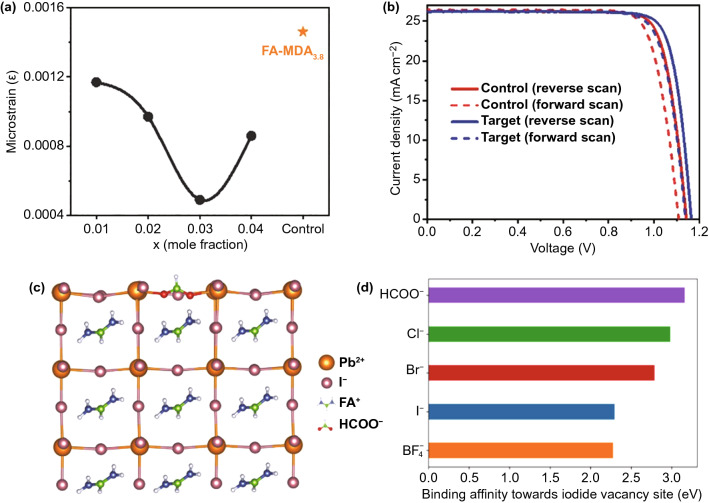
**a** Residual strain calculated in FAPbI_3_ perovskite films doped with different molar ratio (x) of Cs and MDA cations, and the control sample treated with 3.8 mol% MDA. **b** The best *J -V* curves of the control device and the PSCs treated with Cs and MDA. Reproduced with permission from Ref. [27] Copyright 2020 AAAS. **c** Calculated crystal structure demonstrating the passivation of I^−^ vacancy at FAPbI_3_ surface by a HCOO^−^ anion. All chemical compounds are shown in ball-and-stick style. **d** The relative binding energy of different anions at the site of I^−^ vacancy at FAPbI_3_ surface. Reproduced with permission from Ref. [29] Copyright 2021 Nature Publishing Group

To maximize the photon-absorption at the UV–vis region, Kim et al. used the inherent bandgap (1.47 eV) of α-phase FAPbI_3_ to increase the photocurrent of PSCs [27]. They replaced the FA cations by a trace amount (0.03 molar fraction) of cesium (Cs) and methylenediammonium (MDA) cations to stabilize the α-phase FAPbI_3_ without changing its inherent bandgap, but improved the UV–vis absorption of perovskite layer. These effects contribute to a high *J*_SC_ of 26.23 mA cm^−2^ for PSCs, very close to the theoretical current limit of about 27 mA cm^−2^ for 1.47 eV-bandgap semiconductors [7]. Moreover, Fig. 1a shows that co-doping of 0.03 mol Cs and MDA cations also relaxes the lattice strain of FAPbI_3_ by over 70% compared to the sample only treated with MDA cation reported in their previous study [28], which prolongs the carrier recombination lifetime and enables a *V*_OC_ increase of 30 mV (Fig. 1b), leading to a high certified PCE reaching 24.4%.

Besides the cation-doping strategies, substitution of X-site halide anions could also significantly affect the optoelectronic properties of FAPbI_3_ perovskites. Jeong et al. introduced an anion engineering strategy that employs the pseudo-halide anion formate (HCOO^−^) to fill the halide vacancy defects located at grain boundaries and surface of perovskite films [29] and to enhance the crystallinity of FAPbI_3_. It is found that the doping of 2% formate anions could enlarge the grain size to about 2 μm, increasing the crystal orientation along (100) and (200) directions that are better for carrier transport, and suppressing the formation of non-photoactive δ-FAPbI_3_ phase. Moreover, the theoretical calculation revealed that formate anions had a larger binding affinity toward iodide vacancy sites compared to other anions like Cl^−^, Br^−^, and BF_4_^−^ owing to the fact that every carboxylate group can form two Pb–O coordination bonds with the lead cations (Fig. 1c, d). As a result, the FAPbI_3_-based PSCs with pseudo-halide treatment attained a record PCE of 25.6% (certified 25.2%) and a *V*_OC_ of 1.19 V.

Another representative work for the improvement in perovskite layers is the fabrication of uniaxial-oriented perovskite layer with millimeter-sized grains via a methylamine gas-assisted crystallization method [30]. In this study, the methylammonium lead iodide (MAPbI_3_) perovskite film was formed by controlling the evaporation rate of MA gas molecules from liquid intermediate phase of MAPbI_3_·xMA in a closed system. The results demonstrated that a slow release rate of MA molecules from the liquid intermediate phase significantly reduced the supersaturation, leading to a low nucleation density that provided more time for growing large MAPbI_3_ grains during thermal annealing process. Consequently, the perovskite grains with about 1 mm-size can be obtained, resulting in a very low trap density of 9.7 × 10^13^ cm^−3^, approaching that of the MAPbI_3_ single crystal [31], and the PSCs with millimeter-sized MAPbI_3_ perovskite grains exhibited a promising PCE of 21.36%.

### Interface Engineering

The interfacial properties between perovskite and charge transport layers play an important role in the charge recombination mechanism of PSCs, which greatly influence the *V*_OC_ and fill factor (FF) of the solar cells [32–34]. Therefore, many methods have been developed to reduce the non-radiative recombination loss at the interface and optimize the series resistance of the passivation layers for efficiency improvement.

**Fig. 2:**
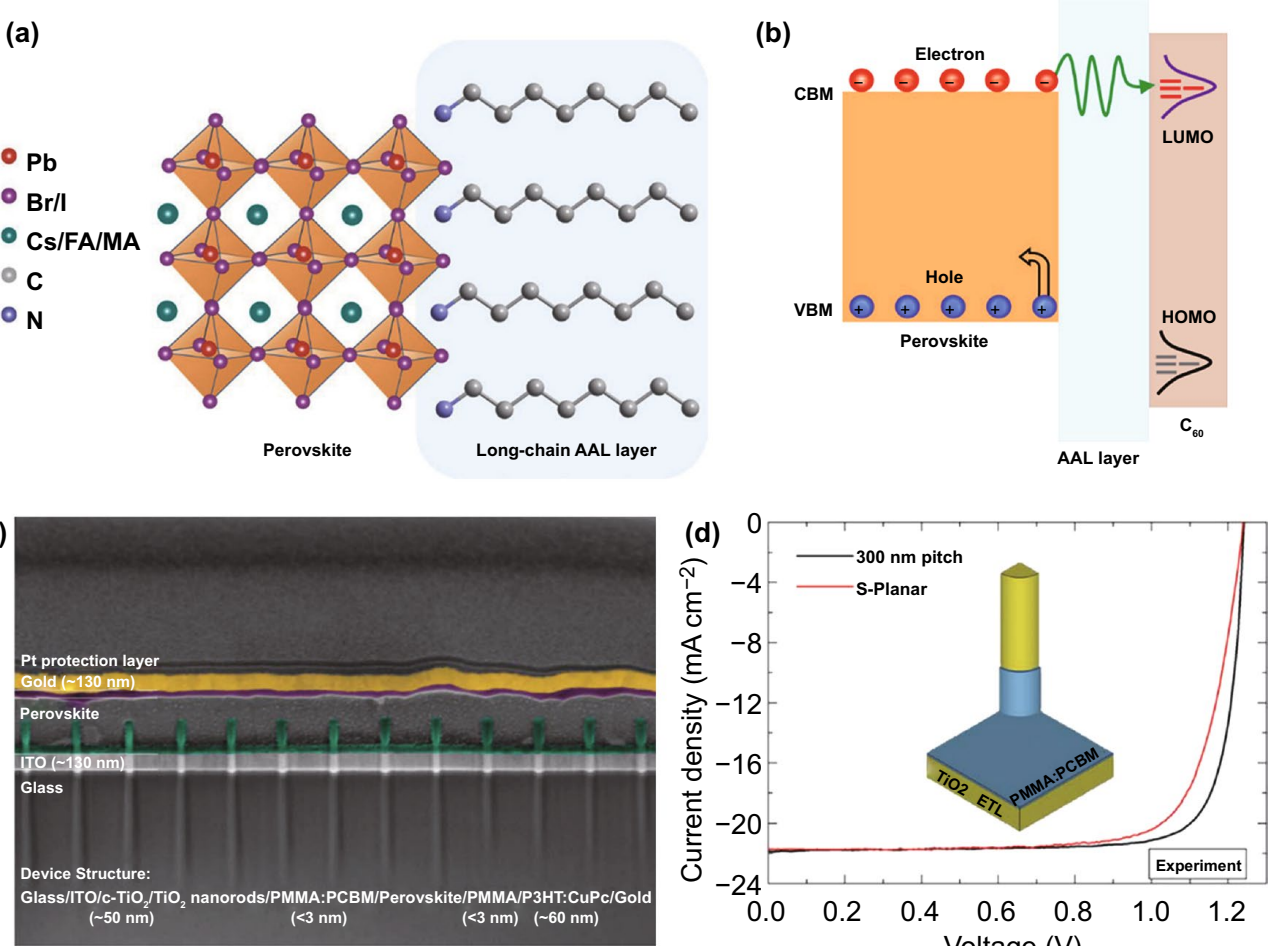
**a** Illustration of the long-chain AALs assembled on perovskite film surface. **b** The electron-tunneling and hole-blocking effects induced by AAL layer at the perovskite/C_60_ interface, VBM and CBM represent the valence band maximum and conduction band minimum of perovskite semiconductor, respectively. Reproduced with permission from Ref. [35] Copyright 2020 Nature Publishing Group. **c** Cross-sectional SEM image of the PSCs designed with TiO_2_ nanorod pattern at the bottom surface of perovskite layer. **d** Experimental *J-V* curves of the PSCs based on planar (reference cell) and the TiO_2_ nanopattern structure, 300 nm pitch represents the TiO_2_ nanorods with a spacing of 300 nm in devices, the inset shows that about 30% of the nanorod is coated with insulating passivation layer. Reproduced with permission from Ref. [37] Copyright 2020 AAAS

To suppress carrier recombination at perovskite surface and grain boundaries, Zheng et al. used a trace amount of surface-anchoring alkylamine ligands (AALs) with different chain lengths for the modification of perovskite surface [35]. They found that the oleylamine ligand with a long alkyl chain showed a more obvious passivation effect for improving carrier recombination lifetime and the *V*_OC_ of PSCs (Fig. 2a), as compared to other organic AALs with shorter alkyl chains, which could be attributed to the electron-tunneling effect in the insulating AALs layers. Such a tunneling effect allows the movement of electrons from perovskite conduction band to the lowest unoccupied molecular orbital (LUMO) of the fullerene-C_60_ layer, but effectively blocks the hole injection from valence band to the highest occupied molecular orbital (LUMO) of C_60_ (Fig. 2b). This passivation technique based on the insulating tunneling layer is also widely applied in silicon solar cells [36]. Based on this oleylamine ligand-anchoring strategy, the inverted-structure PSCs achieved a strong *V*_OC_ improvement up to 110 mV, resulting in a record certified efficiency of 22.3%.

Although the additional passivation layer is beneficial for the *V*_OC_ improvement of PSCs, it may undesirably increase the series resistance of devices and thus leads to the loss of FF. This detrimental effect will become more obvious in the case of large-area devices. In order to balance the improvement of *V*_OC_ and FF, Peng et al. designed a nanoscale localized contact at the polymer-passivated perovskite/TiO_2_ interface to realize a higher FF for 1 cm^2^ PSCs [37]. They deposited a nanopatterned electron-selective TiO_2_ layer via atomic-layer deposition (ALD) to form nanorod-like charge transport channels through the passivated interface (the device structure is shown in Fig. 2c), which provided both effective contact passivation and excellent charge extraction ability for reducing the device series resistance. As a result, they achieved a high FF of 83.9% and a high *V*_OC_ of 1.20 V by employing an ALD TiO_2_ nanopattern with 300 nm spacing at the polymer passivation interface (Fig. 2d), resulting in a record certified PCE of 21.6% for an over 1 cm^2^ cell.

### Charge Transport Layer Design

Rational design of the charge transport layer also boosts the PV parameters of PSCs significantly. Jeong et al. designed two fluorinated isomeric analogs of the well-known hole transport molecule spiro-OMeTAD to modify the energy-level alignment, hydrophobicity, and hole extraction ability in PSCs [38]. They found that the fluorinated group at the meta-position on the benzene ring (spiro-mF) could lower the HOMO position from −4.97 to −5.19 eV and thus provided a more suitable energy-level alignment with the valence band maximum (VBM) of FAPbI_3_ perovskite absorber (-5.40 eV) to reduce the interfacial energy loss, resulting in a *V*_OC_ improvement of the device. Moreover, the F atoms could induce a denser solid-state molecule packing through non-covalent intramolecular interaction, which further improves the electronic contact between spiro-mF and perovskite surface, producing better transport and extraction ability of holes, resulting in a slightly increase of *J*_SC_ and FF. These effects boost the efficiency from 23.44% to 24.84% for PSCs.

**Fig. 3:**
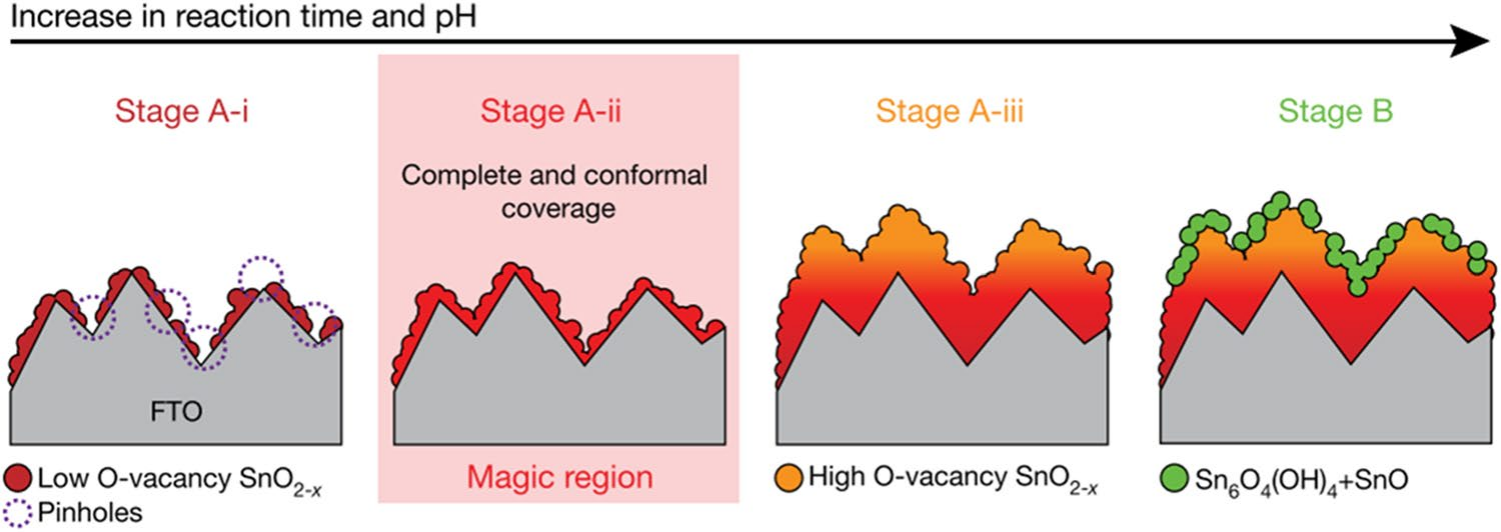
Schematic illustration of four major stages at different reaction time and pH value of the chemical bath deposition for SnO_2_, the magic region highlights the A-ii stage that exhibits an ideal film coverage, morphology and chemical composition of SnO_2_ film. Reproduced with permission from Ref. [39] Copyright 2021 Nature Publishing Group

On the other side, chemical bath deposition was applied to produce a high-quality electron-selective SnO_2_ layer for limiting excess charge carrier recombination in PSCs [39]. Unlike the conventional deposition using SnO_x_ nanoparticle dispersion, chemical bath deposition enables the uniform and complete coverage of SnO_2_ film on the substrate via a rational control of the reaction time and pH value of the SnCl_2_ precursor solution. As can be seen in Fig. 3, at the stage A-i, with a low pH value and short reaction time, the as-deposited SnO_2_ film showed many pinholes. After increasing the pH value to stage A-ii, a low O-vacancy SnO_2_ film (targeted sample) with ideal film coverage, thickness, and chemical composition could be achieved. When further increasing the pH and reaction time to stages A-iii and B, the density of O-vacancy increased significantly and the additional Sn_6_O_4_(OH)_4_ and SnO phases were present, lowering the electron transport ability and inducing charge recombination in SnO_2_ films. By further optimizing the Br concentration in perovskite films, a certified PCE of 25.2% was achieved, corresponding to 80.5% of the theoretical efficiency limit.

## Stability

Stability issues are the major bottleneck for the commercialization of PSCs. The chemical components of metal halide perovskite are bonded through weak interactions such as ionic interaction, hydrogen bonding, and van der Waals forces, which results in the soft-material nature of perovskite semiconductors. Irreversible decomposition of organic species and the ion migration occur easily in PSCs under moisture penetration, continuous light soaking, thermal stress, and external electric field [11, 40–42], which cause damage to both the perovskite and charge transport layers. On the other hand, the leakage of Pb during long-term operation of PSCs under bad weather conditions could further cause environmental and public health risk, which should also be considered as an important stability issue to be addressed for future commercialization. In this part, we discuss the recent research progress on PSC stability from the viewpoint of additive engineering, heterostructure stabilization, and the cell encapsulation technology for reducing lead leakage.

### Additive Engineering

Introducing the additive molecules that could form extra chemical interactions with the volatile components is an effective way to suppress ion migration and irreversible decomposition of perovskite films. Mei et al. [19] introduced bifunctional 5-ammoniumvaleric acid iodide (5-AVAI) to inhibit the methylammonium iodide (MAI) loss on the surface and the crystal reconstruction of MAPbI_3_ grains. 5-AVAI could form hydrogen bonds with the iodine ions through amino groups (−NH_3_^+^), while their carboxyl groups (-COOH) form a strong hydrogen bond with another 5-AVAI in the adjacent grains, which results in a cross-linking of the perovskite surface and interface and a large enhancement of the thermal stability. Besides, non-volatile 5-AVAI at the interfaces limits the volatilization of organic components, which inhibits the forward process of decomposition reaction of MAPbI_3_. It can also stabilize the metal oxide matrix (ZrO_2_ and TiO_2_) via the anchoring effect between -COOH and metal cations, thus restricting ion migration of MA^+^ and I^−^ at the grain boundaries and the interfaces. After encapsulation with a hot-melt polymer film, the printable PSCs treated with 5-AVAI successfully passed the main items of IEC61215:2016 qualification tests, especially working for more than 9000 h at a maximum power point of 55 ± 5 °C without obvious decay.

Furthermore, an organic ionic salt called 1-butyl-1-methylpiperidinium tetrafluoroborate ([BMP]^+^[BF_4_]^−^) was developed to suppress the light-induced phase segregation and degradation in thermally stable CsFA-based lead-halide perovskites [43]. The results indicated that small amounts of [BMP]^+^[BF_4_]^−^ additives could penetrate the entire volume of perovskite film and passivate both the bulk and surface defect states via ionic interaction, leading to a large photovoltage enhancement for the PSCs. During the aging process, [BMP]^+^[BF_4_]^−^ prevented the segregation of Br-rich FAPbBr_3_ phase in mixed-halide perovskite film and suppress the corrosion of silver electrode (one of the key reasons for device degradation) induced by I_2_ generation under light and heat. I_2_ is mainly formed by the combination of interstitial I^−^ and holes, or two neutral iodine atoms [44]. In the presence of [BMP]^+^[BF_4_]^−^, the ion diffusion channels at the grain surface were effectively reduced, resulting in the inhibition of I_2_ formation. Consequently, under full-spectrum simulated sunlight in ambient atmosphere, the unencapsulated and encapsulated PSCs retained 80% and 95% of their post-burn-in efficiencies for 1010 h (60 °C) and 1200 h (85 °C), respectively.

Other additives such as lead chloride, organic dye molecules, phosphorus-based Lewis acid, and cyano derivatives have been reported to suppress ion migration and passivate surface ionic defects in perovskite film, resulting in the improvement of long-term stability [45–47].

### Heterostructure Stabilization

**Fig. 4:**
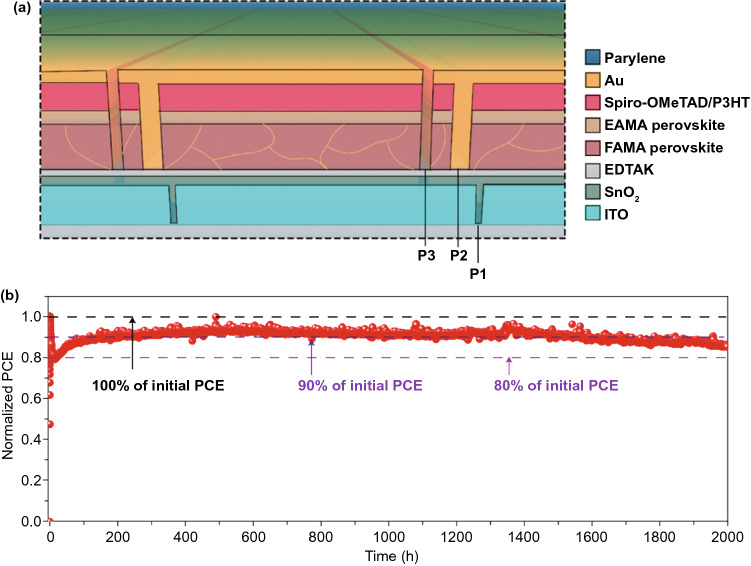
**a** The holistic method containing four treatments for the functional layers and their interfaces in device, including the use of EDTAK to modify SnO_2_ layer, the use of ethylammonium iodide (EAI) to form EAMA-based perovskite atop the FAMA-based perovskite absorber, doping P3HT into the spiro-OMeTAD layer, and using parylene to encapsulate the PSC module. **b** The operational stability of parylene-encapsulated solar module with a structure of SnO_2_-EDTAK/perovskite/spiro-OMeTAD-P3HT/Au measured under continuous 1-sun illumination. Reproduced with permission from Ref. [47] Copyright 2020 Nature Publishing Group

The heterostructures formed between perovskite and charge transport layers play a significant role in the long-term stability of the whole device, so much attention has been paid to stabilizing the interface of each functional layer in PSCs. Liu et al. employed a holistic strategy to stabilize the front and back interface of perovskite layer under operational condition [48].They used ethylenediaminetetraacetic acid dipotassium salt (EDTAK) to treat the electron-selective SnO_2_ surface for stabilization of the front heterostructure (Fig. 4a). The Lewis acid–base reaction between the alkylamine group of EDTAK and Pb^2+^ cations efficiently passivated the vacancy defects in the perovskite film. Besides, incorporation of EDTAK also shifted the conduction band minimum (CBM) of SnO_2_ from −3.69 to −3.95 eV, resulting in a better energy-level alignment for charge extraction. The perovskite/spiro-OMeTAD heterostructure was stabilized by the surface ethylammonium iodide (EAI) modification to form an EAMA-based perovskite capping layer with better ambient stability. Regarding the spiro-OMeTAD/electrode interface, a small amount of P3HT was incorporated to inhibit the degradation caused by inward migration of gold into the perovskite layer and also enhance the moisture stability of spiro-OMeTAD. By employing such a holistic stabilization approach, the PSC modules without encapsulation achieved an efficiency of 16.6% with a designated area of 22.4 cm^2^, and the encapsulated solar modules with parylene-coated cover glass retained approximately 86% of the initial performance after continuous operation for 2000 h under AM1.5G light illumination (Fig. 4b).

Our previous work employed CsFA-based perovskite with high thermal stability as the absorber to construct a scalable integrated heterostructure for PSC modules [49]. Under the operational condition, the iodide ions can easily migrate from perovskite to the hole transport layer (HTL) and change its semiconducting properties from p‐type to n‐type, causing deterioration of hole extraction in PSCs. To stabilize this heterostructure, a scalable bridge‐jointed graphene oxide (BJ-GO) layer was deposited on the perovskite surface to block iodide migration. During this process, 3‐aminopropyl triethoxysilane (APTES) was used to connect the small-size GO nanosheets by forming C-N covalent bonds with GO, resulting in a full‐coverage hydrophobic blocking layer on perovskite surface for inhibiting ion migration, passivating surface under-coordinated Pb^2+^, and protecting perovskite layer from the damage of water in ambient environment. By employing the BJ-GO layer in the PSCs with undoped HTL, we achieved a high PCE of 16.21% for a 36 cm^2^ solar module. The encapsulated module retained over 91% of its initial efficiency after the damp heat test at 85 °C and 85% relative humidity for 1000 h, while maintaining 90% of the initial value for 1000 h under standard operational condition at 60 °C.

### Cell Encapsulation

In 2020–2021, researches on PSC encapsulation mainly focused on reducing the Pb leakage from a broken device. During the long-term operation process, Pb^2+^ showing weak chemical interaction with other perovskite components could also escape from perovskite layer after water penetration, resulting in additional environmental problems. For a typical lead perovskite absorber with a thickness of 550 nm, the unit-area lead concentration is estimated to be about 0.75 g m^−2^, which is more than 100 times higher than that of the commonly used Pb-containing paints (0.007 g m^−2^) [50].

The physical encapsulation with Pb^2+^-absorption materials can effectively suppress the irreversible outflow of Pb from damaged devices into the underground water or soils at severe weather conditions [51]. Li et al. deposited the lead-absorbing materials on both front and back sides of PSCs to reduce the leakage of lead [23]. On the front glass side, they used a transparent Pb-absorbing molecule with phosphonic acid groups that showed a large binding energy with Pb^2+^ cations to absorb lead in water when water seeps into the device. On the back side, they coated a polymer film mixed with lead-coordination agents between the metal electrode and the encapsulation layer. As a consequence, the lead-absorption effects on both sides of PSCs could sequestrate more than 96% of lead leakage in water induced by severe device damage while retain the structural integrity of solar cells.

**Fig. 5:**
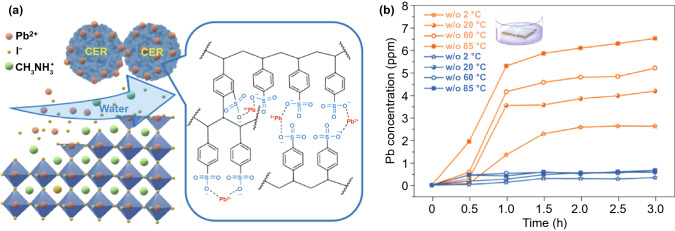
**a** Illustration of how CERs prevent the lead leakage via strong ionic interaction between Pb^2+^ cations in broken perovskite films and the sulfonate groups. **b** Pb-soaking test results in water for the damaged perovskite solar modules without (yellow line) and with (blue line) the CER coating layer on glass side. Reproduced with permission from Ref. [50] Copyright 2020 Nature Publishing Group

Furthermore, a low-cost and chemically robust cation-exchange resin (CER)-based method was developed to prevent lead leakage from broken PSC modules [52]. The results showed that CER exhibited both high adsorption capacity and high adsorption rate of Pb^2+^ in water due to the high binding energy between sulfonate-terminated groups and the common divalent metal ions like Pb^2+^, Ca^2+^, and Mg^2+^ in the mesoporous polymer matrix (Fig. 5a). Additionally, mixing the CER with carbon electrode and integrating them on the front glass of PSC modules have a negligible detrimental impact on the device PCE while reduces lead leakage from mini-modules in water by 62-fold to 14.3 ppb as compared to that of the device without CER (Fig. 5b). The theoretical calculation indicates that the CER treatment could further reduce the lead leakage of large-area perovskite solar panels to lower than 7.0 ppb even under the worst condition that all the PSC sub-modules are broken.

## Perovskite-Based Tandem Solar Cells

The tunable bandgap of ABX_3_ perovskite absorber from 1.2 eV to 3.0 eV benefits the design of silicon-perovskite or perovskite-perovskite tandem devices [34] that enables the theoretical efficiency beyond the Shockley–Queisser limit of single-junction solar cells. For silicon-perovskite tandems, exploiting the ideal wide-bandgap perovskite materials (1.6–1.7 eV) with suitable spectrum response matching the 1.1 eV-bandgap silicon absorber is key to obtaining high efficiency [53]. By contrast, suppressing the generation of Sn^4+^ defects and increasing the carrier diffusion length in narrow-bandgap mixed Sn–Pb perovskites (1.1–1.2 eV) are currently the major tasks for efficient all-perovskite tandem devices.

### Silicon-Perovskite Tandem Structure

In the past two years, the efficiency of silicon-perovskite tandems boosted rapidly from about 25% to over 29% due to the reduced *V*_OC_ deficit of wide-bandgap perovskite top cell enabled by compositional engineering for a stable phase and the selection of charge transport layers [54–56]. Xu et al. [57] reported a triple-halide alloys containing chlorine, bromine, and iodine to tailor the carrier lifetime and suppress the light-induced phase segregation in wide-bandgap perovskite films. They found that direct incorporation of large amounts of Cl (more than 15%) into the double-halide perovskite could produce a uniform halide distribution throughout the film with an ideal bandgap (1.67 eV) matching the spectrum of the bottom 1.12-eV Si absorber. This effect increased the photocarrier mobility by two times and efficiently suppressed the phase separation that was previously found in most of the mixed-halide perovskite compositions [58, 59]. By extending the double-halide to triple-halide components, a distinct efficiency enhancement for opaque single-junction PSCs from 18.15% to 20.42% was demonstrated, with a *V*_OC_ increasing over 100 mV. Moreover, they obtained a PCE of 27.04% in 1-cm^2^ two-terminal monolithic tandems via integrating the optimized perovskite top cells with the silicon bottom cells.

**Fig. 6:**
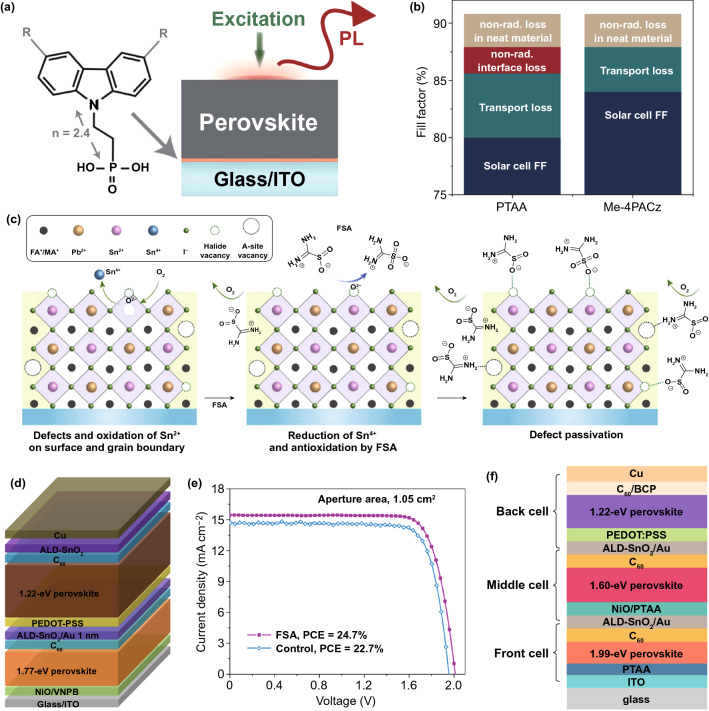
**a** Schematic of the photoluminescence measurement and the chemical structure of carbazole-based HTL molecules, with R representing a methyl group for Me-4PACz. The number of 4 denotes the number of carbon atoms between the phosphonic anchoring group and the carbazole unit for Me-4PACz. **b** The FF loss mechanisms for PTAA and Me-4PACz PSCs based on the detailed balance analysis. Reproduced with permission from Ref. [10] Copyright 2021 AAAS. **c** Schematic illustration of suppressing Sn^2+^ oxidation and the passivation of halide and cation vacancy at the grain surface of mixed Sn–Pb perovskite films enabled by FSA molecule. A-site represents the organic monovalent cations in the lattice. **d** Structure of all-perovskite tandem device with 1.22 eV narrow-bandgap absorber and 1.77 eV wide-bandgap absorber. **e** *J-V* curves of the best control and FSA tandem devices with an aperture area of 1.05 cm^2^. Reproduced with permission from Ref. [9] Copyright 2020 Nature Publishing Group. **f** Device configuration of the all-perovskite triple-junction tandem device. Reproduced with permission from Ref. [65] Copyright 2020 American Chemical Society Publications

Besides the modification of perovskite layer, a novel self-assembled, methyl-substituted carbazole monolayer (Me-4PACz, the chemical structure is shown in Fig. 6a) was developed as the HTL to minimize the non-radiative recombination loss at the hole-selective contact in wide-bandgap PSCs [10], resulting in a record efficiency of 29.15% for Si-perovskite tandem devices. The carbazole unit provided fast hole extraction from the wide-bandgap perovskite layer and the methyl substitution was considered to passivate the defects on perovskite surface, minimizing the FF loss induced by series resistance (transport loss) and the interfacial non-radiative recombination in PSCs (Fig. 6b), as investigated by intensity-dependent absolute photoluminescence measurements. By using the Me-4PACz hole-selective layer, the FF of single-junction PSCs greatly increased from 79.8% (control cell based on PTAA) to 84.0%, and the device *V*_OC_ also showed a large improvement from 1.19 V to 1.25 V.

Textured crystalline-Si subcells have also been reported for Si-perovskite tandem devices to reduce the device reflectance and increase the photon usage rate. Hou et al. [60] directly deposited the solution-processed micrometer-thick perovskite top cell on a fully textured Si-heterojunction bottom cell to fabricate tandem devices. They addressed the carrier-extraction issue in micrometer-thick perovskite layer by increasing the depletion width at the bases of silicon bottom cell and employing a self-assembled 1-butanethiol passivation layer on the perovskite surface. This strategy not only increased the carrier diffusion length but also stabilized the wide-bandgap perovskite phase, enabling a certified efficiency of 25.7%. Similarly, Chen et al. used a nitrogen-assisted blading process to deposit hole transport layer and high-quality planarizing perovskite absorber that fully covered the rough silicon pyramids [61]. Moreover, a textured light-scattering layer was added to the perovskite top cell to reduce reflectance at the front surface, leading to an efficiency of 26.0% for textured Si-perovskite tandem devices.

### All-Perovskite Tandem Structure

Compared to the Si-perovskite tandems, all-perovskite tandems exhibit additional advantages of low materials and fabrication costs because the bottom and top cells can be fabricated by the same preparation process without using specific equipment for other types of solar cells. The initial two-terminal all-perovskite tandems used MAPbBr_3_ and MAPbI_3_ as the wide-bandgap (2.30 eV) and narrow-bandgap (1.55 eV) absorbers, respectively, giving an efficiency of 10.8% [62].

After a few years of development, the efficiency of all-perovskite tandems has increased to over 24% via tailoring the bandgap alignment in devices. The mixed Sn–Pb perovskites with a minimized bandgap of about 1.2 eV are a promising candidate as the narrow-bandgap absorbers [63]. However, the spontaneous oxidation of Sn^2+^ to Sn^4+^ and Sn vacancy cause a high defect density in Sn–Pb perovskites and thus a shorter carrier diffusion length compared to that of the full-Pb counterpart, which lowers the charge transport efficiency in the thick narrow-bandgap absorber (over 1 μm). To overcome this challenge, Lin et al. used metallic Sn to reduce the Sn^4+^ impurities in perovskite precursors via a comproportionation reaction (Sn + Sn^4+^  → Sn^2+^) [64], reducing the defect density from 1.4 × 10^16^ to 5.4 × 10^15^ cm^−3^ and thereby prolonging the diffusion length from 0.75 to 2.99 μm in MA_0.3_FA_0.7_Pb_0.5_Sn_0.5_I_3_ perovskites. After integrating this narrow-bandgap Sn–Pb PSCs (1.22 eV) with the wide-bandgap PSCs (1.77 eV), they obtained a high PCE of 24.8% for the tandem solar cells.

To further reduce the defect density in mixed Sn–Pb perovskite, Lin et al. employed a strongly reductive surface-anchoring molecules, formamidine sulfinic acid (FSA), to reduce the Sn^4+^ defects and passivate the surface ionic vacancy [9]. As shown in Fig. 6c, the sulfinic acid group could react with oxygen molecules and thus suppress the Sn^2+^ oxidation. Meanwhile, the O atoms of sulfinic group serving as an electron donor could form coordination bond with the under-coordinated Sn^2+^ or Pb^2+^ cations to passivate the surface halide vacancy. On the other hand, the formamidine group of FSA showing similar structure with FA cation could also passivate the surface A-site vacancy defects. Such synergistic effect of the surface-anchoring FSA improved the carrier recombination lifetime in Sn–Pb perovskites by three times. Via employing this narrow-bandgap absorber into the monolithic tandem structure (Fig. 6d), they obtained a *V*_OC_ enhancement of about 60 mV and a large PCE improvement from 22.7% to 24.7% for a 1.05-cm^2^ all-perovskite tandem (Fig. 6e).

Furthermore, the solution-processed all-perovskite triple-junction tandem device with a PCE of over 20% was also reported [65]. This triple-junction solar cell contained a 1.99−eV perovskite at the front cell, a 1.60−eV perovskite at the middle cell, and a 1.22−eV perovskite at the back cell (Fig. 6f). By developing compatible interconnecting layers with those solution-processed perovskite absorbers, a high *V*_OC_ of 2.8 V can be achieved, much higher than the value of all-perovskite double-junction solar cells.

### Lead-Free PSCs

With the continuous movement of PSCs to commercialization, the toxicity of Pb element in perovskite absorber arouses the concern of environmental problems [66]. In recent years, a growing number of studies have aimed at developing the eco-friendly lead-free PSCs to directly avoid the use of lead in metal halide perovskite layers. So far, a number of lead-free perovskites based on tin (Sn), antimony (Sb), bismuth (Bi), titanium (Ti), germanium (Ge), and copper (Cu) have been exploited for the application of solar cells [67–71].

Among all the lead-free perovskite materials, the most promising candidate is tin perovskite. Sn has a similar outer electronic structure (ns^2^ np^2^) and ionic radius to Pb, enabling the complete replacement of lead in perovskite lattice without causing notable phase segregation. Furthermore, tin perovskites demonstrate some additional advantages: (1) ideal bandgap close to Shockley–Queisser limit (1.3 ~ 1.4 eV), (2) low exciton binding energy (29 meV for MASnI_3_ and 62 meV for MAPbI_3_), and (3) high charge carrier mobility (*μ*_e_ (electron mobility) = 2000 cm^2^ V^−1^ s^−1^ for MASnI_3_ and 60 cm^2^ V^−1^ s^−1^ for MAPbI_3_) [72, 73].

After a few years of development, the efficiency of tin PSCs underwent a rapid growth from 6 to 11%–13% owing to the careful design of antioxidant additives, mediating the crystallization rate, design of suitable A-site cations such as phenethylammonium (PEA) and pentafluorophen-oxyethylammonium (FOE), and constructing the oriented low-dimensional perovskite structure [22, 74, 75]. Currently, researchers mainly focus on the defect passivation and selection of suitable electron transport layers to minimize the *V*_OC_ loss in tin PSCs.

**Fig. 7:**
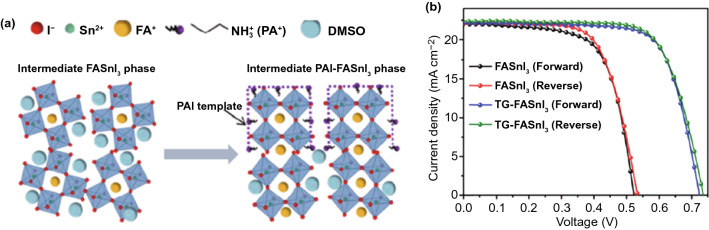
**a** Possible mechanism of the template-growth process for FASnI_3_ perovskite film. **b** *J-V* plots of the champion tin PSCs based on control and template-growth FASnI_3_ perovskite absorbers. Reproduced with permission from Ref. [24] Copyright 2020 The Royal Society of Chemistry

Nishimura et al. combined the bulk ethylammonium (EA) doping and surface edamine passivation to reduce the defect density of FASnI_3_ perovskite films by as much as 1 order of magnitude [21], which resulted in a high device *V*_OC_ of 0.84 V. Furthermore, our group developed a template-growth technique assisted by an n-propylammonium iodide (PAI) salt to reduce the bulk defect density in the solution-processed FASnI_3_ films [24]. The PAI post-treatment increased the electron diffusion length from 70 to 180 nm and thereby reduced the recombination loss in FASnI_3_ absorbers due to the increased crystal orientation along (h00) directions (Fig. 7a), which enabled a significant *V*_OC_ enhancement of 200 mV and a record certified PCE of 11.22% for tin PSCs (Fig. 7b). On the other side, Jiang et al. utilized indene-C_60_ bis-adduct (ICBA) with a high LUMO position as the electron transport layer to reduce the energy-level offset in tin PSCs [76]. They found that a shallower LUMO of −3.74 eV in ICBA than that (−3.91 eV) of traditional [6, 6]-phenyl-C_61_-butyric acid methyl ester (PCBM) can upshift the quasi-Fermi level of electrons, approaching the CBM (−3.69 eV) of FA_0.85_PEA_0.15_SnI_3_ perovskite (PEA represents cation) and thus improving the quasi-Fermi level splitting and the maximum attainable *V*_OC_. Consequently, the ICBA-based tin PSCs obtained an ultra-high open-circuit voltage of 0.94 V, about 300 mV increasing compared to that of the PCBM-based cell, which enables a high PCE of 12.4%. The detailed PV parameters of tin PSCs reported in 2020–2021 are summarized in Table 1.

**Table 1:** Photovoltaic parameters of the tin PSCs reported in 2020–2021, all the device efficiencies were measured in the third-party institutes or accredited test centers

Perovskite	*J*_SC_ (mA cm^−2^)	*V*_OC_ (V)	FF (%)	PCE (%)	Test Center	Refs
FASnI_3_	21.14	0.61	73.4	9.39	AIST^*a*^ (Japan)	[72]
Cs_0.1_FA_0.9_SnI_3_	22.21	0.64	70.8	10.08	Newport (USA)	[75]
PEA_0.15_FA_0.85_SnI_3_	17.40	0.94	75.0	12.40	SIMIT^*b*^ (China)	[76]
FOE_0.02_FA_0.98_SnI_3_	21.95	0.64	72.5	10.16	Newport (USA)	[74]
FA_0.75_MA_0.25_SnI_3_	23.65	0.69	68.0	11.16	KISTEC^*c*^ (Japan)	[73]
PA_0.2_FA_0.8_SnI_3_	22.01	0.70	73.3	11.22	Newport (USA)	[24]

^*a*^AIST represents National Institute of Advanced Industrial Science and Technology

^*b*^SIMIT represents Shanghai Institute of Microsystem and Information Technology, Chinese Academy of Science

^*c*^KISTEC represents the Kanagawa Institute of Industrial Science and Technology

Besides the tin PSCs, other types of lead-free PSCs based on wide-bandgap Bi and Sb perovskites also showed a large efficiency progress. Hu et al. fabricated the bulk-heterojunction perovskite active layers consisting of phase-separated Cs_3_Bi_2_I_9_ and Ag_3_Bi_2_I_9_ to improve the grain orientation and interface band alignment [77], achieving a record efficiency of about 3.6% with a high *V*_OC_ reaching 0.89 V for Bi-based PSCs. Singh et al. used the indacenodithiophene-based organic acceptor with Lewis base groups to improve the morphology of wide-bandgap Cs_3_Sb_2_I_9_ perovskites and enhance the electron-extraction ability in Sb-based PSCs [78], yielding a PCE of 3.25% for the inverted-structure device.

## Conclusions and Perspectives

In 2020 and 2021, researchers from all over the word have reported exciting progress on the improvement of efficiency and stability of PSCs, and both the solar cells and modules have been demonstrated to pass the 85 °C/85% RH test. Furthermore, the entrepreneurs in China and other countries are pushing forward the large-scale production of PSCs. Microquanta Semiconductor has established a 5GW-capacity pilot manufacturing facility for perovskite solar products, which is the first production facility for perovskite PV in the world. At the same time, GCL New Energy is building its 100 MW-capacity production line in Kunshan for fabricating the 18%-efficiency PSC modules with a decrease of 70% in production cost compared to that of the crystalline-Si. Oxford PV has recently increased the efficiency of perovskite-silicon tandem cells to 29.52% and announced that a 125 MW-capacity production line in Berlin is under construction for producing the perovskite-based residential rooftop panels. Moreover, Wonder Solar Ltd. continues to develop the fully printable PSC modules and has demonstrated a 110 m^2^ power generation system outdoor. We hope that such collaboration between academia and PV industry can be further intensified to boost the commercialization of PSCs.

**Fig. 8:**
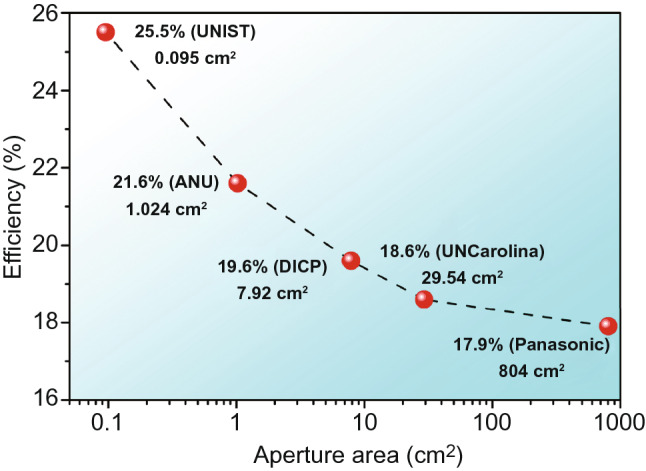
The record efficiency of PSCs as a function of the aperture area in 2020. UNIST represents Ulsan National Institute of Science and Technology (Republic of Korea), ANU represents the Australian National University (Australia), DCIP represents Dalian Institute of Chemical Physics (China), UNCarolina represents the University of North Carolina at Chapel Hill (USA)

Although many efforts have been done, the significantly reduced efficiency upon solar module area scaling-up is still the main challenge to face for the commercialization of PSCs. As shown in Fig. 8, the PCE decreases to 19.6% when the aperture area increases from 0.1 cm^2^ to about 10 cm^2^, and further drops to 17.9% with the area approaching 1000 cm^2^ for PSCs, which still lags far behind that of the crystalline silicon cells (26.7% at 79.0 cm^2^ and 24.4% at 13,177 cm^2^) [6]. Therefore, intensive works should be conducted to precisely control the uniformity of the crystallization process in large-area perovskite films. Also, the fundamental mechanisms relative to the PCE loss in PSC modules should be further studied. On the other side, realizing open-air and high-speed coating of perovskite films is also important for reducing the module manufacturing cost in production line, and some related studies have been done. Deng et al. first demonstrated a tailoring solvent coordination method to blade large-area perovskite films with a high speed of 5.9 m min^−1^ under ambient condition. They used the low-volatile, coordinating solvents for Pb^2+^ components to obtain high-quality perovskite films, yielding a module efficiency of 16.4% [5]. Recently, Rolston et al. reported an open-air rapid spray plasma processing method that enabled a record coating speed of 12 m min^−1^ without any post-annealing for CsFA-based perovskite films, which resulted in a module efficiency of 15.5% [79].

Furthermore, recent study showed that organic solvents used in the mass production of PSC modules such as N,N-dimethylformamide (DMF), N,N-dimethylacetamide (DMAC), N-methyl-2-pyrrolidone (NMP), and gamma-butyrolactone (GBL) are toxic to the human reproductive systems [80]. Therefore, development of green solvent systems or the solvent-free deposition technology for fabricating large-area perovskite film will be an important research topic in the future [81, 82].

Besides the efficiency, more and more attention has been paid to the long-term stability of PSC modules. In 2020, Microquanta company announced that its mass-produced PSC module has passed the strict stability test according to the International Electro technical Commission (IEC) standards, the 20 cm^2^ perovskite module underwent 3000 h damp heat test without degradation, and showed an efficiency loss less than 2% after UV preconditioning test, associated with a product lifetime of over 25 years. Also, Utmo Light Ltd. reported that their PSC mini-module passed the IEC test with a stabilized efficiency of over 20%, which is a milestone for PSCs toward the practical use. Recently, Okinawa Institute of Science and Technology Graduate University (OIST, Japan) reported over 1100-h operational lifetime for a 10 × 10 cm^2^ solar module [83]. Although many research groups and companies claimed that their devices have passed IEC standard test, there are still some stability issues needed to be addressed at the next stage, one important thing is the cell encapsulation technology. A well-designed encapsulation of PSCs should not only block the outside moisture and oxygen effectively, but also suppress the escape of volatile products and lead from the decomposed organic hybrid perovskite layer [84]. In this regard, we believe that a growing number of studies will move to exploit such multifunctional encapsulation materials in the future.

So far, a series of stability tests custom-made for PSCs have been proposed [85], and we therefore suggest a standardized stability test for PSCs and encourage the researchers to measure the module stability at an authorized third-party test center to increase the credibility of their results.

Development of highly efficient lead-free PSCs is also an alternative choice to extend their application range in the PV markets, especially for the indoor power generation like wearable power sources that have a strict limit on lead content [86–88]. It was demonstrated that tin PSCs could be the next generation of PSCs for realizing over 20% efficiency and the strategies for their mass production have been investigated [20]. Moreover, developing reducing solvent system for Sn^2+^ precursor, constructing antioxidant capping layer during the crystallization of tin perovskite film, and the investigation of compatible device encapsulation approaches also led to a large stability improvement of tin PSCs [89, 90]. In our opinion, the application of tin PSCs for indoor PV products could be prior to lead PSCs when their efficiencies reach over 15%.
